# Investigating Environmental Determinants of Injury and Trauma in the Canadian North

**DOI:** 10.3390/ijerph110201536

**Published:** 2014-01-28

**Authors:** Agata Durkalec, Chris Furgal, Mark W. Skinner, Tom Sheldon

**Affiliations:** 1 Frost Centre for Canadian Studies and Indigenous Studies, Trent University, 1600 West Bank Drive, Peterborough, ON K9J 7B8, Canada; 2 Indigenous Environmental Studies Program and Health, Environment, and Indigenous Communities Research Group, Trent University, 1600 West Bank Drive, Peterborough, ON K9J 7B8, Canada; E-Mail: chrisfurgal@trentu.ca; 3 Geography Department, Trent University, 1600 West Bank Drive, Peterborough, ON K9J 7B8, Canada; E-Mail: markskinner@trentu.ca; 4 Environment Division, Nunatsiavut Government, P.O. Box 70, Nain, NL A0P 1L0, Canada; E-Mail: tom_sheldon@nunatsiavut.com

**Keywords:** unintentional injury, search and rescue, Inuit, climate change, sea ice, arctic, environmental health

## Abstract

Unintentional injury and trauma rates are disproportionately high in Inuit regions, and environmental changes are predicted to exacerbate injury rates. However, there is a major gap in our understanding of the risk factors contributing to land-based injury and trauma in the Arctic. We investigated the role of environmental and other factors in search and rescue (SAR) incidents in a remote Inuit community in northern Canada using a collaborative mixed methods approach. We analyzed SAR records from 1995 to 2010 and conducted key consultant interviews in 2010 and 2011. Data showed an estimated annual SAR incidence rate of 19 individuals per 1,000. Weather and ice conditions were the most frequent contributing factor for cases. In contrast with other studies, intoxication was the least common factor associated with SAR incidents. The incidence rate was six times higher for males than females, while land-users aged 26–35 had the highest incidence rate among age groups. Thirty-four percent of individuals sustained physical health impacts. Results demonstrate that environmental conditions are critical factors contributing to physical health risk in Inuit communities, particularly related to travel on sea ice during winter. Age and gender are important risk factors. This knowledge is vital for informing management of land-based physical health risk given rapidly changing environmental conditions in the Arctic.

## 1. Introduction

The preventable death in 2012 of Burton Winters, an Inuit adolescent who perished after becoming lost the sea ice in Labrador, Canada, resonated widely with Canadians, making national headlines as people expressed their sadness and outrage [1]. Despite the increase in public attention on travel safety and search and rescue (SAR) in Canada’s Arctic, and ongoing reports from northern communities about increasing injuries related to environmental changes [2,3], there are still major gaps in our knowledge of factors contributing to land-based injury and trauma in the North (meaning occurring on land, ice, or water). Understanding these determinants is critical for mitigating unintentional injury and trauma and may help prevent deaths like this from occurring in the future. 

For much of the year, Inuit in the circumpolar Arctic use a network of sea ice routes to access wildlife species that are critical to diets and livelihoods, and places that are filled with cultural meaning [4,5]. These uses translate into sea ice being beneficial for health and wellbeing [6]. However, negative physical health impacts can be sustained through experiences such as cold exposure and falling through the ice [7,8]. The public health context of concerns around land-based safety is that mortality rates from unintentional injuries are already disproportionately high in Inuit regions: From 1999 to 2003, the age-standardized mortality rate from unintentional injuries was 4.3 times higher in Inuit Nunangat (Inuit regions in Canada) than Canada as a whole [9]. Rates of drowning for Indigenous Canadians are six times higher than for non-Indigenous Canadians, and eight times higher for snowmobile-related drownings [10,11]. Hospitalization rates for unintentional injuries from land transportation in high Inuit population areas are greater than in high First Nations areas, high Metis areas, and low Aboriginal population areas in Canada for adults, children, and youth [12,13]. Reducing unintentional injuries and drownings related to poor ice conditions has been identified as an important strategy for addressing the disparity in injury rates between Inuit and non-Inuit Canadians [14,15]. 

The reasons for disproportionate risk of unintentional injury among Inuit are numerous and complex. While increased land-based activities among Inuit in a challenging physical environment may lead to increased exposure to environmental hazards, social determinants of health including lower family incomes, lower education, access to culturally-appropriate healthcare and injury prevention programming, and impacts of colonial processes on culture, knowledge, and relationships to the land also play a role in higher unintentional injury rates [10,16,17]. Nonetheless, limitations in injury epidemiology data for Indigenous Canadians, including for Inuit [18,19,20], mean that major gaps in our understanding of the relationships between environmental influences and injury and trauma in northern communities remain. 

Investigating this relationship has become more urgent, as Inuit communities have recently been reporting concerns about increasing accidents and anxiety associated with changing ice and weather conditions and their ability to predict the safety of environmental conditions prior to travel or hunting [2,3]. Changes in sea ice strength and extent and the timing of freeze-up and break-up in the Arctic have been well documented [3,21,22]. However, a limited body of literature has explored the impact of changing sea ice conditions on travel safety [17,23]. 

To explore the role of environmental determinants for injury and trauma in northern communities, this study investigated the role of environmental and other factors in SAR incidents in Nain, Nunatsiavut in Labrador, using a collaborative community-based approach. We examined (1) the frequency of incidents and changes in frequency over time; (2) social and environmental factors associated with or contributing to incidents; and (3) health impacts of incidents.

This project originated with the concern in Canadian Arctic communities regarding increasing unintentional injuries and trauma, and anxiety associated with changing ice and weather conditions, including reports from the community of Nain [2,3,24,25]. Sea ice safety was identified as a priority research area by the Nunatsiavut Government (NG) related to recent unusually mild winter conditions with implications for land use and travel safety. Interest in addressing this issue by the NG and Nain Ground Search and Rescue (NGSAR) and ongoing relationships led to the collaboration on this project.

## 2. Methods

### 2.1. Project Design

We conducted a case study using a mixed methods approach [26,27]. This paper reports results from both SAR data analysis and interpretation, and key consultant interviews with SAR representatives. Ethics approval for this research was granted by Trent University’s Research Ethics Board and the NG Research Advisory Committee.

### 2.2. The Case Study: Nain, Nunatsiavut

**Figure 1:**
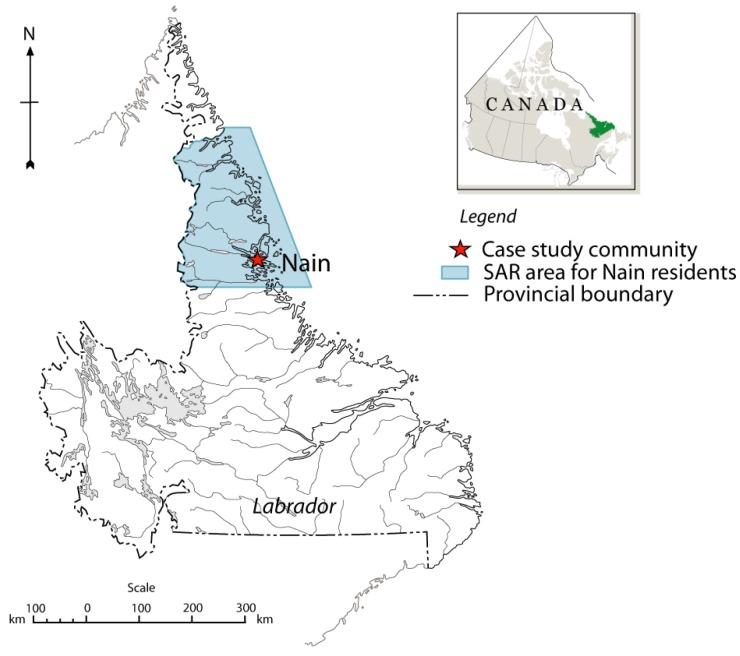
Location of Nain in Labrador, Canada, and the maximum SAR area for Nain residents [31].

**Figure 2:**
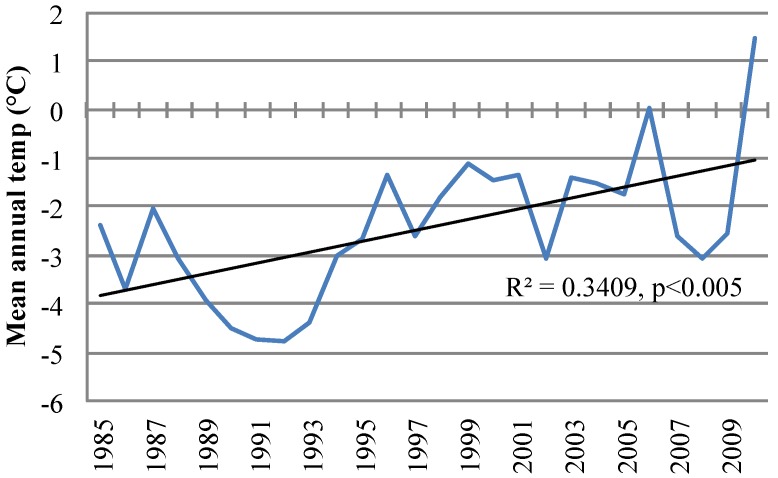
Mean annual temperature in Nain from 1985 to 2010 [32].

The community of Nain is the northernmost community on the east coast of Labrador (56°32′N, 61°41′W) in the Labrador Inuit Settlement Area of Nunatsiavut (Figure 1). The population of the town was 1,188 in 2011, with 92% of the population identifying as Aboriginal [28,29]. Nain is a fly-in community located on an inlet on the Atlantic Ocean protected by islands and surrounded by hilly terrain. The climate of the area is classified as sub-arctic. Environmental activities are important for the traditions, culture, livelihoods, and health of residents [3,4,25]. Climate data indicate a significant upward trend in the mean annual temperature in Nain from 1985 to 2010 (Figure 2). 

### 2.3. Data Collection and Analysis

Before data collection was initiated, we made a preliminary trip to Nunatsiavut in February 2010 to plan the study and build relationships. In July and November 2010, we conducted semi-directed key consultant interviews with two NGSAR members in current and former positions of leadership and two Royal Canadian Mounted Police (RCMP) members in the Nain detachment [30]. Participants were selected using a snowball method [30]. Interviews were recorded by digital audio recorder or notetaking and compensation was provided. Additionally, three meetings were held with NGSAR members in November 2010, March 2011, and May 2011 to share study progress and obtain feedback on analysis. Interviews were transcribed and analyzed using thematic content analysis using QSR International’s NVivo 8 software [30]. Themes were based on interview topics. 

Document review of SAR records from three sources was conducted [26]. We were provided access to NGSAR records, meeting minutes, and notes by the team in July 2010. Fifty-two cases were initially identified spanning 1995 to 2006. We summarized and transformed NGSAR and RCMP records into one quantitative database for analysis in Microsoft Excel. This included variables describing factors contributing to case events (i.e., reasons for SAR assistance). NGSAR members assisted with data cleaning (e.g., merging duplicate cases, filtering false alarms), yielding a total of 40 cases from 1995 to 2007. According to NGSAR members, this represents 50 to 75% of incidents in this time period. Names of 76 individuals were included in the case files; this information was used by a former NGSAR member to place individuals into gender and age categories for case characterization and analysis. 

We filed a request with the RCMP under the Access to Information Act in October 2010 seeking access to missing persons occurrence reports for searches, rescues, or recoveries carried out or authorized by the RCMP; occurring outside the town limits of Nain; and occurring between November 1 to June 15 for the years 2005 to 2010. Reports from before 2005 were deleted by the RCMP and were no longer accessible. Files for 11 cases were received in December 2010. 

We received federal SAR data for Labrador inland (search area 060) and Labrador offshore (search area 009) from 1995 to 2009 from the federal Fisheries and Oceans Canada (DFO) in January 2010. DFO manages federal SAR data. In total there were 116 cases for Labrador inland and 406 cases for Labrador offshore from 1995 to 2009, excluding false alarms and aeronautical incidents. Identifying information had been removed from all case data. The area of interest was identified with the help of NGSAR members, and 85 cases were identified for this area (Figure 1). Commerical fishing, marine transportation, and medical evacuation cases were excluded, yielding 38 cases total for 1995 to 2009. 

Data from the three sources were merged into a single database and cross-checked for duplication. For simplification, cases appearing in both NGSAR and RCMP sources are attributed to NGSAR, and cases in NGSAR or RCMP and DFO sources are attributed to NGSAR or RCMP, respectively. Statistical analyses were conducted in Excel. Data for Nain on land-based travel participation were not available, so estimations of incidence rates were calculated based on the percentage of the Inuit adult population in Nunatsiavut that harvested country foods in 2005 according to gender and age [33], and the total Aboriginal population in Nain in 2006 [28].

## 3. Results

### 3.1. The Context of SAR in Nain

As reported by NGSAR and RCMP consultants, the volunteer-based Nain search and rescue team (NGSAR) is the main group that carries out SAR operations in the Nain area and is mainly active during the winter season. The group began in the early 1990s in response to tragic incidents on the land and the desire to improve SAR response time and effectiveness. The RCMP in Nain helps coordinate and sanctions searches. In some cases, the RCMP will contact the federal Joint Rescue Coordination Centre (JRCC) in Halifax, Nova Scotia for assistance—one of three federal SAR centres in Canada jointly operated by the Department of National Defence (DND) and the Canadian Coast Guard (CCG) [34]. 

### 3.2. SAR Trends

**Figure 3:**
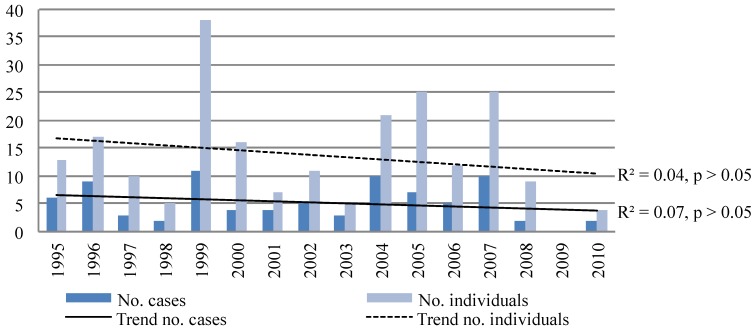
Number of SAR cases and individuals assisted per year in Nain from 1995 to 2010.

SAR data retained for analysis in this study included 49 cases representing 113 individuals handled by local authorities in the Nain area between 1995 and 2010. DFO records indicated that an additional 34 federal cases representing 105 individuals took place between 1995 and 2009 in the same area. Taken together, the data show 83 cases representing 218 individuals occurring in the Nain area between 1995 and 2010. These data represent an estimated average annual incidence rate of 19 individuals per 1,000 (Table 1). There were no significant trends in the number of cases or individuals assisted over time (Figure 3). 

**Table 1:** Number of individuals in SAR cases and estimated annual incidence rate for SAR involvement of individuals from 1995 to 2010 for the Aboriginal population in Nain by gender and age.

Group	Number of Individuals	Estimated Avg. Annual Incidence Rate per 1000
*Gender*		
Females	11	2
Males	71	12
*Age*		
15–25	12	6
26–35	24	14
36–45	12	7
46–55	7	5
56 and over	10	8
*Total*	*218*	*19*

The reason for SAR initiation was reported for 54% (N = 45) of cases. A report of a traveller being overdue was the most frequent mode of SAR initiation; this was the reason for initiation in 78% of cases where the reason was known. The activity of the individuals in distress was unknown in over 43% of cases. For the remainder, the activities that were identified the most frequently (30% of cases each) were hunting and collecting wood for fuel. RCMP consultants reported that most searches are initiated at night after people have been reported overdue by their families, but usually the search will not commence until the morning. An NGSAR consultant reported that most searches take place on the weekend because of higher frequency of travel by people on weekends due to weekday work obligations.

Gender was identified in 38% (N = 82) of cases, primarily from NGSAR records. For these cases, 87% of the individuals assisted were male, 13% were female (Table 1). There was a significant deviation from an equal distribution based on gender (df = 1, N = 82, X^2^ = 43.90, *p* < 0.0005). The SAR incidence rate was also six times higher for males than females (Table 1). The ages of 31% (N = 67) of individuals assisted were identified, and for those individuals, over half were 21 to 40 at the time they were assisted. For the age groups 15–25, 26–45, 45–65, and over 66, there was a significant deviation from a distribution based on Nain population data (df = 3, N = 65, X^2^ = 18.98, *p* < 0.0005). Further, the age group of 26 to 35 had the highest annual incidence rate for SAR involvement (Table 1). There was also a significant downward trend in the frequency of receiving assistance for the age group 15–25 over time (R^2^ = 0.29, *p* < 0.05), while there was no significant trend for the age groups 26–45 (R^2^ = 0.01, *p* > 0.05) and over 45 (R^2^ = 0.02, *p* > 0.05). According to an NGSAR consultant, individuals assisted are typically male, which is corroborated by SAR data. It was reported that travellers in their twenties will use Global Positioning Systems (GPSs) and travel in groups so can help each other in case of challenges, while the people NGSAR assists tend to be middle-aged male travellers who have gone hunting by themselves and have broken down or encountered other obstacles. However, this is not clearly reflected in the SAR data, where the frequency of individuals in need of help was 32% lower for those over 40 compared with the under 40 group.

Some indicator of health status was included in 29% (N = 24) of cases involving 62 people, all from NGSAR or RCMP records. Those receiving assistance were described as “fine” or “okay” in 66% of these cases, and as experiencing some trauma or injury in the remaining 34% of cases, with all of these incidents occurring during the ice-travel season. Individuals were reported as tired because of extreme exertion from walking long distances in 8% of cases and hungry in 8% of cases. Individuals were exposed to extreme or prolonged cold in 25% of cases. Two-thirds of these individuals, or 8 people, were reported as nearly perishing from freezing or experiencing severe frostbite, while one perished from cold exposure. Four percent of cases resulted in drowning after falling through the ice. There was no significant trend in the frequency of health impacts over time (R^2^ = 0.04, *p* > 0.05). 

**Figure 4:**
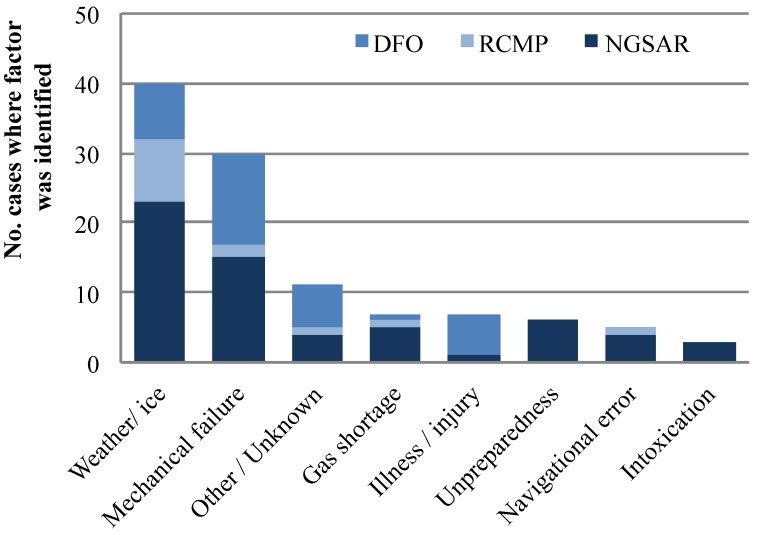
Contributing factors for Nain SAR cases from 1995 to 2010.

The majority of cases from DFO records took place between June and October, as 76% of cases from DFO records were boating-related (Figure 4). However, 96% (N = 47) of the incidents handled on a local level by NGSAR and the RCMP took place from November to May, during the time of year when residents typically travel by ice, with 53% of cases taking place in February and March. These data corroborate information from key consultants, who reported that most searches take place in winter, from January to March in particular.

Weather and ice conditions were the single most frequent contributing factor for cases, contributing to 58% of cases in NGSAR records, 100% of cases in RCMP records, and 24% of cases from DFO records (Figure 4). However, there was no significant trend in the number of cases (R^2^ = 0.01, *p* > 0.05) or percentage of cases (R^2^ = 0.15, *p* > 0.05) where weather/ice were contributing factors over time. Specifically focusing on the critical ice season, there were also no significant trends over time in the number of cases (R^2^ = 0.006, *p* > 0.05), number of individuals assisted (R^2^ = 0.04, *p* > 0.05), or number of cases where weather/ice were factors (R^2^ = 0.09, *p* > 0.05). NGSAR consultants reported that most searches are on the sea ice as that is the main route of travel, and that causes of most searches tend to be blizzard conditions, running out of gas, or snowmobile breakdown. Further, it was reported that there are typically many snow storms in March that contribute to SAR cases, and that as the weather becomes milder in spring, snowmobile engines tend to overheat and this may contribute to mechanical breakdowns. This observation complements results that show where weather or ice conditions were a factor, mechanical failure was also a contributing factor 25% of the time, more than any other secondary factor. Further, it was reported that a typically higher occurrence of fog at this time of year than in other seasons may cause navigational error. 

## 4. Discussion

This study investigated the role of environmental determinants for injury and trauma in a Canadian Inuit community, using SAR incident and injury data spanning 1995 to 2010 and interview data with SAR representatives. SAR data show 83 cases involving 218 individuals from 1995 to 2010 in the Nain area. These data represent an estimated average annual incidence rate of 19 individuals per 1,000 (Table 1). Data showed that weather and ice conditions were the single most frequent contributing factor for cases, and that nearly all cases managed locally by NGSAR or the RCMP took place during the ice season. These results indicate that environmental influences are critical factors contributing to health risk in Inuit communities, and that land-based health risk is associated with the use of sea ice in the winter months in particular. Given the limited nature of data on injury epidemiology for Inuit, very little is understood about environmental influences on injury and trauma in northern communities [18,19,20]. In the Nunavik Inuit Health Survey, 13% of injuries reported resulted from snowmobile use [19]. However, we do not have cause-specific injury data for Nunatsiavut, as no questions on injuries were asked in the Inuit Health Survey in Nunatsiavut. Given the disproportionately high rates of unintentional injuries among Inuit, these results create a strong basis for conducting further investigation into the relationship between environmental influences and unintentional injury in northern communities [9]. 

Results demonstrate no significant trends over time in the number of cases or individuals assisted per year from 1995 to 2010, number of cases or individuals assisted during the ice season over time, or number or percentage of cases where ice and weather were a contributing factor over time. These results may indicate that (1) changes in weather and ice conditions have not had a significant impact on land-based incidents, contrary to local perception [3,25,35]; (2) SAR data was not sufficiently sensitive to detect impacts from changing environmental conditions on experiences of injury and trauma reported in communities thus far, possibly due to the small sample size; or (3) environmental and social or other factors are offsetting each other, such that clear trends in SAR events perceived by community residents in association with changing environmental factors are not discernable. Based on the perception and reports of increasing accidents related to changing ice and weather conditions in Inuit communities, we suggest that the latter two interpretations are more likely to be accurate [2,3]. This raises important questions about the underreporting of incidents of injury and trauma related to changes in ice and weather conditions, and the role of other factors that may be influencing land-based safety or SAR practices. For example, adaptations for increasingly hazardous conditions, such as increased knowledge gathering, preparation, and risk-sharing by travelling in groups [24,35,36], could mean that additional incidents are being managed independently or with informal assistance from family and friends, and not resulting in increasing SAR events thus far. It is worth noting that travel risk management practices are mediated by social factors in complex ways. For instance, adoption of new technologies like GPSs can be beneficial for mitigating risk, especially when paired with existing travel knowledge and skills, but there is also concern in Inuit communities that it can encourage risk-taking behavior stemming from an increased sense of security [2].

Our results also show that 34% of individuals (N = 21) sustained minor to severe health impacts during winter travel in cases where health status was indicated, including three deaths. This rate of physical impact is concerning, particularly because these data represent only a portion of impacts sustained between 1995 and 2010 by Nain residents when travelling on the ice and land. First, this is related to inconsistent recording of health status in NGSAR and RCMP records and limited health information (mortality rates only) in DFO records, resulting in the underreporting of morbidity. Second, our data set does not capture all SAR incidents between 1995 and 2010 as the NGSAR data set represents only 50%–75% of incidents in which NGSAR assisted; RCMP data is missing before 2005; and we have no way to confirm that all relevant Missing Persons cases were identified and released by the RCMP. Third, many incidents on the land and ice in Nain are managed without SAR assistance. Key consultants reported that the majority of incidents on the land and ice are managed by travellers independently or with support from friends and family from the community. Given these issues of underreporting, we argue that these data represent a small portion of the actual injury and trauma burden sustained by Inuit in Nain related to travel on sea ice, and that the potential future influence of changing environmental conditions on injury and trauma remains poorly understood. 

Results of this study also demonstrate that age and gender are important risk factors for SAR incidents, which corresponds to injury mortality and morbidity risk factors for drownings and off-road vehicle collisions in the Northwest Territories (NWT) in Canada [18]. Males are a major risk group for drowning deaths related to snowmobiling, non-motorized activities on the ice, and boating in Canada [11]. In our study, male travellers were six times more likely to need SAR assistance than female travellers, and the estimated annual incidence rate also demonstrates a six times higher likelihood of SAR-involvement by males (Table 1). Other studies have explored different but flexible gendered roles during travel and hunting in Inuit communities (e.g., hunters and trip leaders are typically men) [37]. These results indicate that gendered travel and hunting practices in Nain translate into differential health risk experiences. Further, over half of individuals in cases from NGSAR and RCMP records where age was identified were 21 to 40. The age group of 26 to 35 also had the highest estimated annual incidence rate (Table 1). The Canadian Red Cross found that the peak incidence rate for snowmobile-related drowning deaths in Canada among 25 to 34 year olds [11]. However, there was a significant downward trend in the frequency of receiving assistance for those 15–25, possibly because of decreased participation in hunting by this age group or shorter trips [24]. Intoxication was the least common factor associated with SAR incidents, being identified as a contributing factor in only three cases. This result contrasts with other health risk research for northern communities, as intoxication was identified as one of the most important risk factors for injury in the NWT [18]. Alcohol was a contributing factor in 23% of traffic crash-related injuries between 1991 and 2001 in NWT [38], while in Nunavik, 32% of ATV or snowmobile drivers over 15 surveyed reported having driven under the influence of drugs or alcohol in the year prior to the survey [19]. Further, alcohol was a factor in 59% of ice-related snowmobile drownings in Canada [11]. However, participant observation conducted during the larger study, of which this paper is a part, indicates that there may be place-based differences in alcohol consumption patterns in town compared with being on the land [35]. This potential explanation indicates the need to consider how place influences the role of risk factors for injury and trauma. 

We acknowledge gaps in each data set that contribute to there likely being more SAR cases and incidents of land-based injury and trauma during the time period covered by this study than we have been able to identify. The necessity of relying on regional data to estimate incidence rates is also a limitation. However, by demonstrating these gaps in existing data, our study also underscores the likely chronic and systemic underreporting of land-based incidents and the importance of improving land-based injury surveillance in northern regions. 

## 5. Conclusions

This study contributes to an area of growing health concern where existing research is sparse. Unintentional injury rates are disproportionately high in the North, but our understanding of the role of environment for injury is still limited. This study shows that environmental influences, particularly weather and ice conditions, are the leading cause of SAR events in an Inuit community and the key factors contributing to health risk during land-based travel. Further, it extends previous literature on the importance of gender and age as determinants of land-based injury and trauma. Looking forward, we strongly advocate for investigations that examine the underreporting of land-based injury and trauma in Arctic communities, given concerns about increases in injuries due to rapidly changing ice and weather conditions. 
